# Evaluation of Epigenetic Age Acceleration in Growth Hormone (GH)-Deficient Children After 6 Months of Recombinant Human GH Replacement Therapy: Anti-Ageing GH vs. Pro-Ageing Insulin-like Growth Factor 1 (IGF-1)?

**DOI:** 10.3390/jcm14113840

**Published:** 2025-05-29

**Authors:** Antonello E. Rigamonti, Valentina Bollati, Chiara Favero, Benedetta Albetti, Adele Bondesan, Nicoletta Marazzi, Silvano G. Cella, Alessandro Sartorio

**Affiliations:** 1 Department of Clinical Sciences and Community Health, Dipartimento di Eccellenza 2023–2027, University of Milan, 20129 Milan, Italy; silvano.cella@unimi.it; 2 EPIGET Lab, Department of Clinical Sciences and Community Health, Dipartimento di Eccellenza 2023–2027, University of Milan, 20122 Milan, Italy; valentina.bollati@unimi.it (V.B.); chiara.favero@unimi.it (C.F.); benedetta.albetti@unimi.it (B.A.); 3 Occupational Health Unit, Fondazione IRCCS Ca’ Granda Ospedale Maggiore Policlinico, 20122 Milan, Italy; 4 Experimental Laboratory for Auxo-endocrinological Research, Istituto Auxologico Italiano, Istituto di Ricovero e Cura a Carattere Scientifico (IRCCS), 28824 Piancavallo-Verbania, Italy; a.bondesan@auxologico.it (A.B.); sartorio@auxologico.it (A.S.); 5 Experimental Laboratory for Auxo-endocrinological Research, Istituto Auxologico Italiano, Istituto di Ricovero e Cura a Carattere Scientifico (IRCCS), 20145 Milan, Italy; n.marazzi@auxologico.it

**Keywords:** growth hormone deficiency, replacement therapy, recombinant human growth hormone, short stature, chronological age, biological age, epigenetic age, age acceleration, DNA methylation, IGF-1

## Abstract

**Background:** One of the most debated topics in experimental and clinical endocrinology is the impact of hypo- and hyper-somatotropism on the extension/shortening of the lifespan, the results of experimental, clinical, and epidemiological studies being extremely conflicting. Biological age, a surrogate of lifespan, can be measured through different methods, including the age-related epigenetic modifications of DNA. **Objective:** The present study aimed to evaluate the biological (epigenetic) age and age acceleration in a group of growth hormone (GH)-deficient (GHD) children (F/M = 5/5; age: 11.0 ± 2.7 years), treated with recombinant human GH (rhGH) for 6 months at a daily dose of 0.025–0.035 mg/kg. **Results:** Treatment with rhGH significantly increased height velocity and circulating insulin-like growth factor 1 (IGF-1) levels. Biological and chronological ages were significantly correlated at baseline and after 6 months of rhGH replacement therapy. Treatment with rhGH reduced age acceleration, an effect that became significant only after adjustment for IGF-1. In a linear regression model for longitudinal data, after adjustment for rhGH treatment, age acceleration was significantly associated with IGF-1 levels, an effect missing when considering the interaction rhGH treatment × age acceleration at 6 months of rhGH treatment. **Conclusions:** (rh)GH, when administered to GHD children, exerts anti-ageing effects, which become evident after removal of the presumably pro-ageing effects of IGF-1.

## 1. Introduction

The role of growth hormone (GH) in the processes of mammalian ageing has been extensively explored in several experimental, clinical, and epidemiological studies, with contrasting results [1].

For a long time, in the context of the geriatric endocrinology, somatopause has been defined as a syndrome that, pathophysiologically due to the age-related decline of somatotropic function, encompasses a series of symptoms and clinical signs, including changes in body composition (such as visceral adiposity and sarcopenia), cognitive deficit, cardiovascular diseases, xerosis, osteoporosis, etc. [2]. Under an evolutionary view, somatopause, which involves both animals and humans, is a “protective” process against the formation of tumours, due to the removal of GH-mediated stimulation of cell proliferation, including the mitogenic effect by insulin-like growth factor 1 (or IGF-1), and against other chronic degenerative or metabolic diseases (i.e., osteoarthrosis or diabetes mellitus) [3].

Experiments in animals demonstrated that GH deficiency and GH resistance, with the ensuing hyposomatotropism, caused by gene mutations that impair adenohypophisial development, GH secretion or GH/IGF-1-mediated receptorial or post-receptorial mechanisms, produce an impressive extension of longevity [4]. Hyposomatotropism reduces or delays, at least in animals, the biological mechanisms underlying cell senescence and, consequently, ageing [5].

In humans, mainly taking into account the results of several clinical and epidemiological studies carried out in subjects affected by Laron’s syndrome (e.g., deletion of the GH receptor gene), the impact of hyposomatropism on longevity remains controversial [1]. On the contrary, there is a unanimous consent about an improved health status in these subjects independently of the age group, which would derive from a (cumulative) decrease in age-related diseases [6].

So, when a condition of hyposomatropism is present in animals, an extension of lifespan can be easily observed, whereas in human beings healthspan is more likely to be shown [7].

Nevertheless, in general population, the advanced age in men and women is associated with low plasma levels of IGF-1, which have not been consistently associated with human longevity [8].

By contrast, GH hypersecretory states, either in animals (e.g., GH releasing hormone [GHRH] transgene mice) or in humans (e.g., acromegalia, which, in the most of cases, is due to a GH-secreting pituitary adenoma), are associated with a dramatically reduced life expectance [9,10]. Supraphysiological circulating IGF-1 levels increase the risk of several diseases, including some tumours (e.g., colon carcinoma), and they also accelerate ageing in serum, as demonstrated by the studies evaluating biological age [11,12].

While the beneficial effects related to the administration of recombinant human GH (rhGH) in GH-deficient (GHD) children and adults are indisputable [13,14], the use of rhGH as “elixir of long life” in the somatopause, for decades extensively studied, can be definitely abandoned. The reason for this choice is the adverse reactions (even severe) associated with this hormone treatment in older people, among which include diabetes mellitus, arterial hypertension, atherosclerosis, and tumours [15].

While the rhGH-induced synchronising effects on bone age in comparison to chronological age have been extensively studied [15], our knowledge about the relationship between GH (and/or IGF-1) and biological age is minimal, mainly in a clinical context [16]. In particular, biological age can be determined with different methodological approaches, including the epigenetic one [17], which might be more affordable due to GH-mediated actions on the biomolecular mechanisms underlying the epigenetic regulation of DNA [18].

In this context, *Ames* mice, characterised by a mutation in the *Prop1* gene, which results in a multiple hormone deficit, including GH function, show a decreased expression in DNA methyltransferase 1 (DNMT1), with increased levels of DNA methyltransferase 3a (DNMT3a), when compared to wild-type animals. This would suggest a role of DNA methylation in the hyposomatotropism-related longevity of this animal model. *Ames* mice also show an increased expression of glycine N-methyltransferase (GNMT), an enzyme that converts S-adenosyl-methionine (SAM) to S-adenosyl-homocysteine and sarcosine. This enzymatic deficit results in decreased levels of SAM, a substrate for all DNA methyltransferases. Therefore, the increased GNMT expression in the *Ames* mouse might depress the typical (hyper-)methylation of CpG sites that is observed with advancing of age, a process phenotypically associated, in the wild-type counterpart, to the increased epigenetic age, but that, on the contrary, when blocked, would contribute to the reduced incidence of tumour in these animals [19].

Based on the previous premises, evaluation of epigenetic (biological) age and age acceleration in GHD children, treated with rhGH, might represent a valuable clinical setting to epigenetically monitor the effectiveness (or harmfulness?) of the treatment, including the synchronisation of chronological bone and biological (epigenetic) ages (or promoting biological ageing?). This biomolecular event (based on DNA methylation) might be used in combination with the standard auxometric evaluation and the determination of circulating levels of IGF-1, which, in the current clinical practice, are performed in the diagnosis and follow-up of the GHD subject.

The aim of the present study was to evaluate epigenetic (biological) age in a group of GHD children before and after 6 months of rhGH treatment and to correlate their epigenetic age acceleration with auxometric and biochemical parameters adopted in the current clinical practice for monitoring this clinical condition.

## 2. Materials and Methods

### 2.1. Subjects and Protocol

Ten children of both sexes (F/M = 5/5), attending the Research Centre for Growth Disorders, Istituto Auxologico Italiano, IRCCS, Milan, Italy, were selected and then recruited to participate in the present study For each of them a diagnosis of isolated GH deficiency was established according to the criteria included in the AIFA note #39 concerning this disease (i.e., short stature ≤ −3 SD or ≤ −2 SD and growth for year ≤ −1.0 SD for age and sex, evaluated after at least 6 months) and GH peak at two different pharmacological stimulation tests < 8 ng/mL). The main exclusion criterion from the present study (and the treatment with rhGH) was the presence of organic diseases at the hypothalamic-pituitary level (through cerebral NMR investigation).

At basal conditions (pre-treatment, T0), demographic, clinical, and anthropometric/auxometric data, including body composition evaluation using the bioimpedancemeter analysis, were collected in all the children. The same work-up was repeated after 6 months of rhGH treatment (T6). Each subject underwent a hormone replacement therapy with rhGH at a daily dose of 0.025–0.035 mg/kg BW [body weight] (or 0.7–1.0 mg/m^2^ BA [body area]).

In fasting conditions (at least 12 h), a blood sample was drawn at T0 (i.e., before starting the hormone replacement therapy with rhGH) and at T6 (i.e., after 6 months of rhGH treatment, about 10–12 h after the last daily administration of rhGH).

The study protocol was approved by the Ethical Committee (EC) of the Istituto Auxologico Italiano, IRCCS, Milan, Italy (EC code: 2023_03_21_09; research project code: 01C317; acronym: ETABIOGHD).

### 2.2. Metabolic Variables

Total cholesterol (T-C), high-density lipoprotein cholesterol (HDL-C), low-density lipoprotein cholesterol (LDL-C), triglycerides (TG), glucose, insulin, and high-sensitivity C-reactive protein (hsCRP) were measured in serum/plasma samples obtained at T0 and T6.

Colorimetric enzymatic assays (Roche Diagnostics, Monza, Italy) were used to determine serum T-C, LDL-C, HDL-C, and TG levels. The sensitivities of the assays were 3.86 mg/dL [1 mg/dL = 0.03 mmol/L], 3.87 mg/dL [1 mg/dL = 0.03 mmol/L], 3.09 mg/dL [1 mg/dL = 0.03 mmol/L] and 8.85 mg/dL [1 mg/dL = 0.01 mmol/L], respectively.

Serum glucose level was measured using the glucose oxidase enzymatic method (Roche Diagnostics, Monza, Italy). The method’s sensitivity was 2 mg/dL [1 mg/dL = 0.06 mmol/L].

Serum insulin concentration was determined by a chemiluminescent immunometric assay, using a commercial kit (Elecsys Insulin, Roche Diagnostics, Monza, Italy). The sensitivity of the method was 0.2 µIU/mL [1 µU/mL = 7.18 pmol/L].

Hs-CRP was measured using an immunoturbidimetric assay (CRP RX, Roche Diagnostics GmbH, Mannheim, Germany). The sensitivity of the method was 0.3 mg/L.

The intra- and inter-assay coefficients of variation (CVs) were the following: 1.1% and 1.6% for T-C, 1.2% and 2.5% for LDL-C, 1.8% and 2.2% for HDL-C, 1.1% and 2.0% for TG, 1.0% and 1.3% for glucose, and 1.5% and 4.9% for insulin.

Plasma IGF-1 levels were determined using a commercial kit for enzyme-labelled chemiluminescent immunometric assay (Mediagnost GmbH, Tuebingen, Germany). The value of sensitivity was 10 ng/mL. Intra- and inter-assay CVs were 3.5% and 7%.

For each patient, the homeostatic model assessment of insulin resistance (HOMA-IR) was assessed according to the following formula: (insulin [μIU/mL] × glucose [mmol/L])/22.5 [20].

### 2.3. Determination of the Epigenetic Age

Subjects provided a blood sample, which was collected in EDTA-containing tubes and immediately stored at −80 °C until assayed for epigenetic age.

Blood samples were thawed, and genomic DNA was extracted using the Wizard Genomic DNA Purification Kit (Promega; Madison, WI, USA) according to the manufacturer’s instructions.

Epigenetic age was calculated considering the DNA methylation (DNAm) pattern of 5 CpG sites at five genes (ELOVL2, C1orf132/MIR29B2C, FHL2, KLF14, TRIM59) as reported elsewhere [21]. The DNA samples (500 ng) were plated at a concentration of 25 ng/μL in plates of 96 wells each and were treated with sodium bisulfite using the EZ-96 DNA Methylation-Gold Kit (Zymo Research; Irvine, CA, USA) following the manufacturer’s instructions and eluted in 200 μL. Then, 10 μL of bisulfite-treated template DNA was added to 25 μL of GoTaq Hot Start Green Master mix (Promega), 1 μL of the forward primer (10 μM), and 1 μL of the 50-end-biotinylated reverse primer (10 μM) to set up a 50 μL PCR reaction. PCR cycling conditions and primer sequences have been previously reported [21].

Biological (epigenetic) age (Y) was calculated as follows:


Y = 3.26847784751817 + 0.465445549010653 × methC7-ELOVL2 − 0.355450171437202 × methC1-C1orf132 + 0.306488541137007 × methC7-TRIM59 + 0.832684435238792 × methC1-KLF14 + 0.237081243617191 × methC2-FHL2

### 2.4. Statistical Analysis

Demographic, lifestyle, biochemical, and clinical characteristics before and after 6 months of rhGH treatment were compared using paired statistical tests. For continuous variables, data were expressed as mean ± standard deviation if normally distributed, and comparisons were performed using the paired t-test. When variables were not normally distributed, data were presented as median [Q1, Q3], and the Wilcoxon signed-rank test for paired data was applied. Categorical variables were reported as frequencies and percentages.

Correlations between chronological age and epigenetic age at baseline and after 6 months of rhGH treatment were assessed using Spearman’s rank correlation coefficient.

Epigenetic age acceleration was computed as the residual from a linear regression of DNAm age on chronological age, representing the deviation of biological age from that expected based on chronological age. This approach yields a measure of epigenetic age acceleration that is uncorrelated with chronological age. Positive values (>0) indicate accelerated epigenetic ageing, meaning the individual appears biologically older than their chronological age.

The associations of age acceleration with the demographic, lifestyle, biochemical, and clinical characteristics were evaluated using linear mixed-effect regression models for paired data. We used mixed-effects multivariable linear regression models adjusted for time (pre vs. post rhGH treatment). A random intercept is assigned to each participant, allowing for individual-specific variations in the baseline age acceleration, which accounts for within-subject correlation over repeated measurements. Afterwards, to assess whether the treatment modified these associations over time, we included an interaction term between treatment status (pre/post rhGH treatment) and the covariates of interest, thereby evaluating the potential effect modification of the intervention.

The statistical analyses and graphic plotting were performed using SAS software (version 9.4; SAS, Cary, NC, USA).

*p*-values below 0.05 were considered statistically significant.

## 3. Results

### 3.1. Comparison of Parameters Before and After rhGH Treatment

Table 1 compares all demographic, biochemical, and clinical parameters in GHD children before and after 6 months of rhGH treatment. In brief, rhGH treatment significantly increased height velocity (HV) and HV standard deviation score (SDS), calculated according to the Italian reference growth charts for age and sex [22], an effect that was congruent with the significant increases in IGF-1. Glucose, insulin, and T-C levels as well as HOMA-IR were significantly higher after 6 months of rhGH treatment.

### 3.2. Chronological vs. Biological Ages Before and After rhGH Treatment

**Figure 1:**
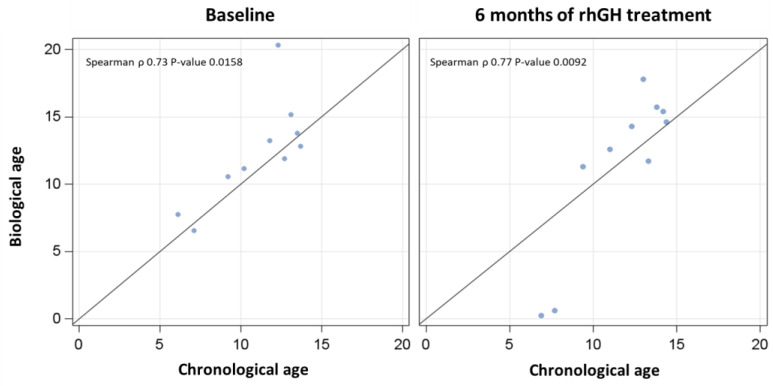
Correlations between biological and chronological ages at baseline (T0—left panel) and after 6 months of rhGH treatment (T6—right panel).

Chronological and biological ages were significantly correlated both at baseline and after 6 months of rhGH treatment (Figure 1).

**Figure 2:**
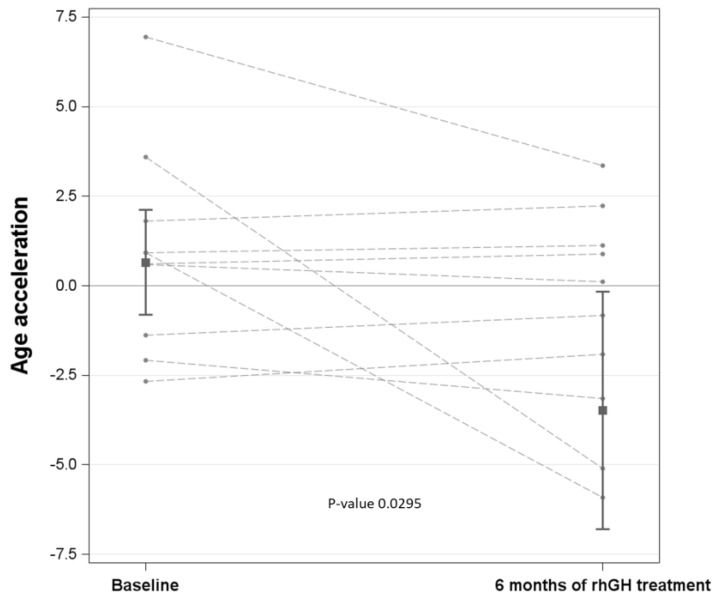
Marginal means of age acceleration for the two times (i.e., baseline vs. 6 months of rhGH treatment), adjusted for IGF-1 levels, with the 95% confidence interval and the *p*-value derived from the comparison. The value of 120 µg/L, corresponding to the median of IGF-1 levels at baseline, was fixed for the adjustment.

Age acceleration was lower after rhGH treatment, an effect that became significant only after adjustment for IGF-1 (Table 2; Figure 2).

### 3.3. Association of Age Acceleration with Each Parameter: Effect of rhGH Treatment

When evaluating the effect modification of rhGH treatment on the association of age acceleration with the other parameters, age acceleration was significantly associated with height (cm), weight (kg), glucose, insulin, and HOMA-IR (Table 3).

### 3.4. Association of Age Acceleration with Each Parameter: Adjustment for rhGH Treatment

When considering “rhGH treatment” as an adjustment factor, age acceleration was significantly associated with height (SDS), glucose, LDL-C, and IGF-1 (Table 4).

## 4. Discussion

In the present study, 6 months of rhGH treatment was highly effective in stimulating growth when administered to a group of GHD children, as evidenced by increased post-treatment HV and IGF-1 levels.

The main finding of the present study was the lower age acceleration, measured through an epigenetics-based algorithm [21], after rhGH treatment. Although this result was not significant, it is noteworthy that the statistical significance was reached after adjustment for IGF-1 levels. In our opinion, this “adjusted” result should not be simply interpreted as due to a reduced sample size or a statistical artefact, but rather as a consequence of a “true” biological phenomenon, presumably a paradoxically opposed action of GH and IGF-1 (see below).

In this regard, in animal models, there is strong and consistent evidence that reduced GH/IGF-1 signalling extends both lifespan and healthspan. The final effects of GH and IGF-1 on life-span or life-health in humans remain controversial because of conflicting results obtained in many experimental, epidemiological, and clinical studies, which have been published for a long time [1]. The controversy primarily lies in human studies, where differently from the animal ones, the effects are more complex, context-dependent, and influenced by factors such as disease states, treatment duration, and baseline hormone levels.

A possible explanation of this debated topic might derive from the present study: GH exerts anti-ageing effects, while IGF-1 has pro-ageing effects. In particular, when considering our GHD children, treated with rhGH for 6 months, the anti-ageing effect of GH (or rhGH) can be evidenced only after removal of the pro-ageing effect by IGF-1 (through the statistical adjustment for IGF-1 in the comparison of age acceleration at baseline vs. 6 months of rhGH treatment, T0 vs. T6). The results of regression models further support our hypothesis (i.e., anti-ageing GH vs. pro-ageing IGF-1). When considering rhGH treatment as an interacting factor, age acceleration is significantly associated with IGF-1 for the effect at T6, but not at T0, though the interaction between rhGH treatment × IGF-1 is not significant, indicating that age acceleration increases (i.e., pro-ageing effect) only after rhGH-stimulated IGF-1 increases. Moreover, when modelling regressions with the adjustment for rhGH treatment, age acceleration was significantly associated with IGF-1, meaning that, removing the anti-ageing effect of GH (or rhGH treatment), only the pro-ageing effect of IGF-1 becomes evident.

Our simple, but surprising conclusion might let any author reviewing the results of previous studies regarding the relationship between GH/IGF-1 function and longevity [1], though it is difficult, even in genetically modified animal models, to separate the effects by GH and IGF-1 because of the hepatic and tissue GH-stimulated production/release of IGF-1, which represents the old “somatomedin hypothesis” [23].

Though the somatomedin hypothesis represents a paradigm in experimental and clinical endocrinology, in more recent studies, GH has been demonstrated to promote growth plate chondrogenesis and longitudinal bone growth with a direct (independent) mechanism, even when the local effects of IGF-1 (and IGF-2, too) are prevented [24]. This represents an example of IGF-independent GH-mediated effects. So, by interacting with specific receptors, namely GH receptor and IGF-1 receptor, GH and IGF-1 might activate distinct intracellular signalling pathways that, respectively, do not or do promote ageing [25,26].

The ultimate molecular mechanisms underlying the anti-ageing/pro-ageing effects of GH/IGF-1 are likely epigenetic [27]. In this regard, GH and IGF-1 have been demonstrated to regulate biochemical machinery involved in epigenetic modifications of DNA, such as DNMT1 and DNMT3a [19].

Our hypothesis of opposed actions of GH/IGF-1 (i.e., anti-ageing/pro-ageing) might re-fuel the long-lasting debate concerning the use of rhGH in the somatopause [2]. In particular, some adverse effects of this claimed “elixir of youth” for elderly people, including atherosclerosis, osteoarthrosis, and tumours, might depend upon IGF-1 (deleterious as endowed with pro-ageing effects) rather than upon GH (beneficial as endowed with anti-ageing effects) [3]. Again, the pharmacological issue is to dissociate GH actions from IGF-1 actions, the latter being hormone-stimulated by the former (somatomedin hypothesis) [23]. Combined administration of rhGH with an anti-IGF-1 monoclonal antibody might, at least in animal models, solve this (intriguing) question.

Before closing, a critical limitation of our study needs to be mentioned. In particular, we are aware of the sexual dimorphism in the control of GH secretion and the biological effects ensuing from exogenous rhGH administration. Unfortunately, GHD is a “rare” disease, and, although our Institution is a reference centre on a national basis, recruiting a vast number of these patients may be a difficult challenge. So, caution should be exercised in interpreting the results of our study before any extrapolation or generalisation. However, regarding this issue, other considerations should be taken into account: (1) our subjects were pre-pubertal or peri-pubertal children; so, differently from adults with GHD, we believe that the sexual dismorphism in rhGH-induced biological effects, including epigenetic age, is somewhat limited; (2) due to the relatively limited sample size (n = 10 subjects), no “conclusive” statistical analyses can be performed by adjusting for sex or evaluating two subgroups (females vs. males); (3) a “balanced” number of female/male children were recruited (i.e., 5/5) in a sort of tentatively biological compensation.

## 5. Conclusions

To the best of our knowledge, the present study, carried out in a group of GHD children treated with rhGH, demonstrates, for the first time, a lower age acceleration, yet after 6 months, an anti-ageing effect that, presumably mediated by GH, becomes evident by removing the IGF-1-induced pro-ageing effect. Further studies are mandatory to investigate the maintenance or change in biological age in—and health consequences on—GHD children treated with rhGH for a long time.

## Figures and Tables

**Table 1:** Demographic, biochemical, and clinical characteristics of the study group (N = 10): comparison between baseline and 6 months of rhGH treatment.

Parameter	Baseline	6 Months of rhGH Treatment	*p*-Value
Age, years	11.0 ± 2.7	11.6 ± 2.6	0.6961
DNAm age, years	12.3 [10.6;13.8]	13.4 [11.3;15.4]	0.6822
Age acceleration, years	0.92 ± 2.8	−0.92 ± 3.1	0.1790
Gender			
Male	5 (50%)	5 (50%)	-
Female	5 (50%)	5 (50%)	
Height, cm	133 [113;139]	138 [119;147]	0.2556
Height SDS	−2.5 ± 0.3	−2.2 ± 0.4	0.0712
Weight, kg	32.0 [19.1;40.1]	35.4 [20.0;45.1]	0.4814
Weight SDS	−2.2 [−2.4;−1.2]	−1.9 [−2.4;−0.8]	0.6288
BMI, kg/m^2^	17.0 [14.9;20.3]	17.7 [14.7;22.6]	0.9109
BMI SDS	−0.8 [−1.5;−0.1]	−0.9 [−1.5;0.5]	1
FM, %	22.7 [15.4;31.2]	21.9 [16.9;28.5]	0.7941
FFM, kg	23 [18.5;27.1]	28.9 [16.4;32.7]	0.2274
HV, cm/year	3.9 ± 1.4	8.7 ± 2.6	<0.0001
HV SDS	−2.8 [−3.8;−1.8]	1.7 [0.1;4.1]	0.0014
IGF-1, µg/L	120.5 [102;182]	341 [203;510]	0.0076
Glucose, mg/dL	81.8 ± 5.9	92.5 ± 6.1	0.0012
HbA1_c_, %	5.0 ± 0.2	5.3 ± 0.2	0.0226
Insulin, mU/L	5.0 ± 3.0	10.8 ± 4.7	0.0039
HOMA-IR	1.1 [0.7;1.3]	2.4 [1.7;2.6]	0.0114
T-C, mg/dL	153 ± 21	177 ± 23	0.0258
HDL-C, mg/dL	52 ± 14	65 ± 19	0.0868
LDL-C, mg/dL	90 ± 16	103 ± 22	0.1727
TG, mg/dL	51 [44;69]	55.5 [47;87]	0.5279
CRP, mg/dL	0.0 [0.0;0.3]	0.0 [0.0;0.1]	0.8796

For normal distribution, values are expressed as mean ± standard deviation, and, when possible, we applied a paired *t*-test. When not normally distributed, values are expressed as median [Q1, Q3], and we used the Wilcoxon signed rank test for paired data. Categorical data are reported as frequencies and percentages.

**Table 2:** Linear mixed-effects regression models for paired data to evaluate the changes in age acceleration between the two times: T6 (6 months of rhGH treatment) – T0 (baseline). The first model was univariate; the second was adjusted for IGF-1.

Univariate Model	Difference	Estimate	95% CI		*p*-Value
Age Acceleration	T6 (6 months of rhGH)–T0 (baseline)	−1.846	−3.843	0.151	0.0701
**Model** **adjusted for IGF-1**	**Difference**	**Estimate**	**95% CI**		***p*** **-value**
Age acceleration	T6 (6 months of rhGH)–T0 (baseline)	−4.137	−7.862	−0.412	0.0295

**Table 3:** Effect modification of rhGH treatment on the association between age acceleration and clinical characteristics. We used linear mixed-effects regression models for paired data with the interaction term rhGH treatment × independent variable.

Independent Variable		β	SE	95% CI		*p*-Value	*p*-Value of Interaction Term “rhGH Treatment × Variable”
Height, cm	effect at a T0	−0.113	0.034	−0.180	−0.047	0.0008	0.0029
	effect at a T6	0.057	0.068	−0.076	0.190	0.3995	
Height, SDS	effect at a T0	−7.247	2.627	−12.396	−2.098	0.0058	0.4599
	effect at a T6	−5.231	2.021	−9.192	−1.270	0.0096	
Weight, kg	effect at a T0	−0.113	0.048	−0.207	−0.019	0.0190	0.0272
	effect at a T6	0.053	0.070	−0.084	0.190	0.4491	
Weight, SDS	effect at a T0	−1.446	0.733	−2.884	−0.009	0.0485	0.1313
	effect at a T6	−0.453	0.784	−1.990	1.085	0.5638	
BMI, kg/m^2^	effect at a T0	−0.212	0.125	−0.458	0.033	0.0902	0.0867
	effect at a T6	0.174	0.218	−0.253	0.602	0.4234	
BMI, SDS	effect at a T0	−0.629	0.425	−1.462	0.204	0.1387	0.2700
	effect at a T6	0.083	0.691	−1.270	1.437	0.9040	
FM, %	effect at a T0	−0.029	0.024	−0.076	0.017	0.2158	0.4662
	effect at a T6	−0.078	0.062	−0.200	0.044	0.2085	
HV, cm/year	effect at a T0	−0.385	0.592	−1.546	0.775	0.5151	0.6195
	effect at a T6	−0.086	0.274	−0.624	0.451	0.7529	
HV, SDS	effect at a T0	0.738	0.896	−1.017	2.493	0.4099	0.3585
	effect at a T6	−0.216	0.276	−0.757	0.325	0.4346	
Glucose, mg/dL	effect at a T0	−0.341	0.096	−0.529	−0.154	0.0004	0.0017
	effect at a T6	0.012	0.117	−0.218	0.242	0.9180	
HbA1_c_, %	effect at a T0	−1.776	3.359	−8.359	4.806	0.5969	0.0608
	effect at a T6	5.714	1.737	2.310	9.117	0.0010	
Insulin, mU/L	effect at a T0	−0.441	0.256	−0.942	0.061	0.0851	0.0197
	effect at a T6	0.239	0.136	−0.027	0.506	0.0786	
HOMA-IR	effect at a T0	−2.273	1.213	−4.650	0.105	0.0610	0.0139
	effect at a T6	0.863	0.478	−0.073	1.800	0.0708	
T-C, mg/dL	effect at a T0	0.049	0.041	−0.032	0.129	0.2361	0.8341
	effect at a T6	0.043	0.039	−0.034	0.119	0.2728	
HDL-C, mg/dL	effect at a T0	−0.057	0.042	−0.140	0.026	0.1813	0.4983
	effect at a T6	−0.021	0.031	−0.082	0.041	0.5093	
LDL-C, mg/dL	effect at a T0	0.103	0.040	0.025	0.181	0.0099	0.3050
	effect at a T6	0.056	0.028	0.001	0.112	0.0475	
TG, mg/dL	effect at a T0	0.082	0.046	−0.008	0.173	0.0748	0.2528
	effect at a T6	0.004	0.047	−0.088	0.097	0.9238	
CRP, mg/dL	effect at a T0	−2.333	2.062	−6.375	1.708	0.2578	0.8097
	effect at a T6	−0.176	10.191	−20.151	19.798	0.9862	
IGF-1, µg/L	effect at a T0	0.010	0.016	−0.022	0.041	0.5483	0.9205
	effect at a T6	0.011	0.005	0.001	0.021	0.0260	

**Table 4:** Association of age acceleration with each parameter, here-in listed. We used linear mixed-effects regression models for paired data adjusted for rhGH treatment.

Independent Variable	β	SE	95% CI		*p*-Value
Height, cm	−0.019	0.040	−0.099	0.060	0.6332
Height SDS	−5.833	1.808	−9.376	−2.291	0.0013
Weight, kg	−0.011	0.043	−0.096	0.073	0.7926
Weight SDS	−0.759	0.590	−1.916	0.398	0.1984
BMI, kg/m^2^	−0.020	0.115	−0.245	0.204	0.8594
BMI SDS	−0.239	0.420	−1.062	0.584	0.5693
FM, %	−0.035	0.024	−0.083	0.013	0.1496
HV, cm/year	−0.125	0.270	−0.655	0.405	0.6440
HV SDS	−0.117	0.278	−0.662	0.428	0.6740
Glucose, mg/dL	−0.176	0.087	−0.346	−0.005	0.0439
HbA1c, %	1.809	2.080	−2.269	5.886	0.3846
Insulin, mU/L	0.059	0.086	−0.109	0.227	0.4914
HOMA-IR	0.257	0.320	−0.371	0.884	0.4223
T-C, mg/dL	0.044	0.037	−0.028	0.116	0.2274
HDL-C, mg/dL	−0.031	0.026	−0.082	0.021	0.2394
LDL-C, mg/dL	0.069	0.025	0.020	0.117	0.0053
TG, mg/dL	0.025	0.038	−0.050	0.099	0.5155
CRP, mg/dL	−2.398	1.815	−5.955	1.159	0.1864
IGF-1, µg/L	0.011	0.005	0.001	0.021	0.0260

## Data Availability

The datasets used and/or analysed in the present study will be uploaded on www.zenodo.org and available from the corresponding author upon a reasonable request.

## Abbreviations

BA: body area; BMI, body mass index; BW: body weight; CRP, C reactive protein; DNAm: DNA methylation; DNMT: DNA methyltransferase; F, female; FFM, free fat mass; FM, fat mass; GH, growth hormone; GHRH, GH-releasing hormone; HBA1_c_, glycated haemoglobin; HDL-C, high-density lipoprotein cholesterol; HOMA-IR, homeostatic model assessment for insulin resistance; HV, height velocity; IGF-1, insulin-like growth factor 1; LDL-C, low-density lipoprotein cholesterol; M, male; SDS, standard deviation score; T-C, total cholesterol; TG, triglycerides.
